# Characterization of a Carbapenem-Resistant *Kluyvera Cryocrescens* Isolate Carrying *Bla*_ndm-1_ from Hospital Sewage

**DOI:** 10.3390/antibiotics8030149

**Published:** 2019-09-16

**Authors:** Ying Li, Li Luo, Zhijiao Xiao, Guangxi Wang, Chengwen Li, Zhikun Zhang, Yingshun Zhou, Luhua Zhang

**Affiliations:** 1 Department of Immunology, School of Basic Medical Sciences, Southwest Medical University, Luzhou 646000, Sichuan, China; 2 Department of Pathogenic Biology, School of Basic Medical Sciences, Southwest Medical University, Luzhou 646000, Sichuan, China

**Keywords:** *Kluyvera*, NDM-1, IncX3, *bla*_KLUC-2_, carbapenemase

## Abstract

Carbapenem-resistant *Enterobacteriaceae* have been a global public health issue in recent years. Here, a carbapenem-resistant *Kluyvera cryocrescens* strain SCW13 was isolated from hospital sewage, and was then subjected to whole-genome sequencing (WGS). Based on WGS data, antimicrobial resistance genes were identified. Resistance plasmids were completely circularized and further bioinformatics analyses of plasmids were performed. A conjugation assay was performed to identify a self-transmissible plasmid mediating carbapenem resistance. A phylogenetic tree was constructed based on the core genome of publicly available *Kluyvera* strains. The isolate SCW13 exhibited resistance to cephalosporin and carbapenem. *bla*_NDM-1_ was found to be located on a ~53-kb self-transmissible IncX3 plasmid, which exhibited high similarity to the previously reported pNDM-HN380, which is an epidemic *bla*_NDM-1_-carrying IncX3 plasmid. Further, we found that SCW13 contained a chromosomal *bla*_KLUC-2_ gene, which was the probable origin of the plasmid-born *bla*_KLUC-2_ found in *Enterobacter cloacae*. Phylogenetic analysis showed that *K. cryocrescens* SCW13 exhibited a close relationship with *K. cryocrescens* NCTC10483. These findings highlight the further dissemination of *bla*_NDM_ through clonal IncX3 plasmids related to pNDM-HN380 among uncommon *Enterobacteriaceae* strains, including *Kluyvera* in this case.

## 1. Introduction

Carbapenem antibiotics are commonly considered as the most effective treatment option for infections caused by extended-spectrum beta-lactamase (ESBL)-producing *Enterobacteriaceae*. However, carbapenemase-producing *Enterobacteriaceae* have become a major threat to public health due to its rapid global dissemination [1]. New Delhi metallo-β-lactamase (NDM) is a type of metallo-β-lactamase that is able to hydrolyze a wide range of clinically available β-lactams (including carbapenems) [2]. Since the initial discovery of *bla*_NDM-1_ from a *Klebsiella pneumoniae* isolate in India in 2008 [3], 24 variants of NDM have been identified thus far [2]. Among them, NDM-1 is the most prevalent one with a worldwide distribution. It has been found in various bacterial species, and has disseminated among humans, food animals, as well as the environment [4,5]. In addition, *bla*_NDM-1_ was found to be carried on a variety of plasmid types [2,6], such as IncX3, IncFIA, IncFIB, and IncA/C, which facilitated the horizontal transfer of *bla*_NDM-1_ among members of *Enterobacteriaceae*, raising a great challenge for clinical treatment.

*Kluyvera* is a group of Gram-negative rod-shaped bacteria that was proposed as a new genus within the *Enterobacteriaceae* in 1981. The *Kluyvera* genus consists of five species: *K. ascorbata*, *K. cryocrescens*, *K. georgiana*, *K. intermedia*, and the novel *K. intestini* [7,8]. Among them, *K. intestini*, *K. ascorbata*, and *K. cryocrescens* were reported to be related to clinical infections in humans [8,9,10]. There are existing reports on the detection of *bla*_KPC-2_ and *mcr-1* in *K. ascorbate* [11,12], *bla*_KPC-2_ in *K. georgiana* [13], as well as *bla*_GES-5_, *bla*_NDM-4_, and *bla*_OXA-48_ in *K. intermedia* [14,15,16]. However, multidrug resistance in *K. cryocrescens* remains rare.

*Kluyvera* is believed to contain the natural progenitor of genes encoding close homologs of CTX-M type β-lactamase, and *K. cryocrescens* is found to be closely related to the CTX-M-1 and KLUC groups [17]. Five *bla*_KLUC_ variants have been determined thus far, which share 77–86% amino acid identity with other CTX-M members [18]. *bla*_KLUC-1_ was demonstrated to be located on the chromosome of *K. cryocrescens* in 2001 [19], and *bla*_KLUC-2_, *bla*_KLUC−3_, *bla*_KLUC−4_, and *bla*_KLUC−5_ were harbored on the plasmids from *Escherichia coli*, *Enterobacter cloacae*, and *K. pneumoniae* [18]. In this study, we characterized one *K. cryocrescens* isolate carrying a plasmid-mediated *bla*_NDM-1_ from hospital sewage in China. Our work determined the complete nucleotide sequence of this NDM-1-producing *K. cryocrescens*, investigated the genetic environment of *bla*_NDM-1_, and performed phylogenetic analysis of *Kluyvera* strains. Furthermore, we confirmed the hypothetical chromosomal counterpart for *bla*_KLUC-2_ previously identified on a plasmid in *E. cloacae*.

## 2. Results and Discussion

### 2.1. Antimicrobial Susceptibility of SCW13

SCW13 was resistant to meropenem (minimum inhibitory concentration (MIC), 128 µg/mL), imipenem (MIC, 256 µg/mL), ceftriaxone (MIC, ≥512 µg/mL), cefotaxime (MIC, ≥512 µg/mL), cefoxitin (MIC, 256 µg/mL), aztreonam (MIC, 512 µg/mL), and fosfomycin (MIC, 256 µg/mL), but susceptible to ciprofloxacin (MIC, ≤4 µg/mL), gentamicin (MIC, ≤4 µg/mL), amikacin (MIC, 32 µg/mL), colistin (MIC, ≤4 µg/mL), and tigecycline (MIC, ≤4 µg/mL) (Table 1). Species identification by 16s rRNA gene sequencing identified SCW13 as *K. cryocrescens*.

### 2.2. Genome Characteristics of SCW13

Draft genome sequence of SCW13 was assembled into 79 contigs (69 were >1000 bp in length) with a 53.4% GC (Guanine and Cytosine) content, which comprised 5,436,727 bp. Average nucleotide identity (ANI) calculation confirmed that SCW13 belonged to *K. cryocrescens*, as it had an OrthoANIu value of 99.23% against the reference strain *K. cryocrescens* NBRC 102467, which is obviously above the 95–96% cut-off usually used to define a bacterial species [20].

Prediction using ResFinder showed that SCW13 had five types of antimicrobial resistance genes mediating resistance to β-lactams (*bla*_NDM-1_, *bla*_SHV-12_, *bla*_CTX-M-3_), quinolones (*qnrS1*), sulfonamides (*sul1*), fosfomycin (*fosA*), and trimethoprim (*dfrA21*, *dfrA14*). Among these resistance genes, *fosA* was located on the chromosome, and the remaining resistance genes were carried by plasmids. Resistance plasmids were completely circularized using PCR and Sanger sequencing. *bla*_NDM-1_ and *bla*_SHV-12_ were carried by a 52,941-bp IncX3 plasmid, which is assigned pNDM1_SCW13 here. *bla*_CTX-M-3_, *qnrS1*, and *dfrA14* were carried by a 54,815-bp IncN plasmid pSCW13-1. *sul1* and *dfrA21* were carried by a 113,711-bp IncFIB plasmid pSCW13-2.

### 2.3. Analysis of the Bla_NDM-1_-Harboring Plasmid PNDM1_SCW13

In the strain SCW13, *bla*_NDM-1_ was able to be transferred to *E. coli* J53 at a frequency of ~10^−4^ (transconjugant/recipient). Compared with the recipient strain J53, transformants containing *bla*_NDM-1_ showed significantly increased resistance to carbapenems and cephalosporins (Table 1), suggesting that *bla*_NDM-1_ in SCW13 was functional and pNDM1_SCW13 was a self-transmissible plasmid.

pNDM1_SCW13 was 52,941 bp in size with an average GC content of 49.28% and contained 66 open reading frames (ORFs); none of the other resistance genes listed above except *bla*_SHV-12_ was co-harbored with *bla*_NDM-1_ on pNDM1_SCW13. A complete nucleotide sequence search against the GenBank database showed that pNDM1_SCW13 was almost identical to previously described IncX3 plasmids pRJA274 (GenBank accession no. KF877335) from *Raoultella planticola* in Shanghai in 2012 [6], and pNDM-HN380 (GenBank accession no. JX104760) from *K. pneumoniae* in Hongkong in 2011 [21], with only two and four single nucleotide polymorphisms (SNPs), respectively. It also showed high similarity (>99% identity) to p128379-NDM (Accession no. MF344560) from *Enterobacter hormaechei*, pZHDC33 (Accession no. KX094555) from *E. coli*, and pABC80-NDM-1 (Accession no. MK372383) from *Citrobacter freundii*. In China, IncX3-type plasmids carrying *bla*_NDM_ variants have been widely found among *Enterobacteriaceae* isolates [2,22,23,24,25]. Our present study further supplements those previous studies and expands the host range to *Kluyvera* spp.

**Figure 1:**
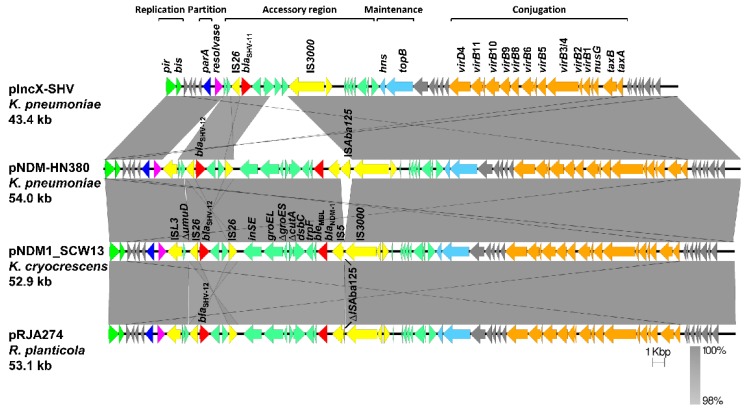
Comparison of linear maps of the *bla*_NDM-1_-carrying IncX3 plasmids. The complete sequence of pIncX-SHV was used as the reference. Open reading frames (ORFs) are shown as arrows to indicate the direction of transcription and are colored in accordance with their predicted gene functions. Homologous segments (representing ≥98% sequence identity) are indicated by light gray shading. Regions are drawn to scale from accession numbers pIncX-SHV (JN247852), pNDM-HN380 (JX104760), and pRJA274 (KF877335). The alignment is a pairwise BLASTn alignment performed using Easyfig.

Figure 1 showed a linear comparison of pNDM1_SCW13 with three other reference IncX3 plasmids. These plasmids had a highly conserved backbone, with sequence polymorphism in the accessory region between *res* and *hns*. Compared to the counterpart on pNDM-HN380 and pRJA274, *bla*_NDM−1_ clusters on pNDM1_SCW13 showed a conserved linear organization (*IS3000*-*Δ*IS*Aba125*-IS*5-Δ*IS*Aba125-bla*_NDM-1_-*ble*_MBL_-*trpF-dsbC-cutA-groES-groL-insE*), whereas the length of the remnant of IS*Aba125* element between IS*3000* and IS*5* varied, with 917 bp on pNDM-HN380, 28 bp on pRJA274, and 33 bp on pNDM1_SCW13. A deletion of 82 bp in the truncated *umuD* gene upstream of SHV-12 was observed on pNDM1_SCW13.

### 2.4. Analysis of the Chromosomally-Encoded KLUC-2

**Figure 2:**
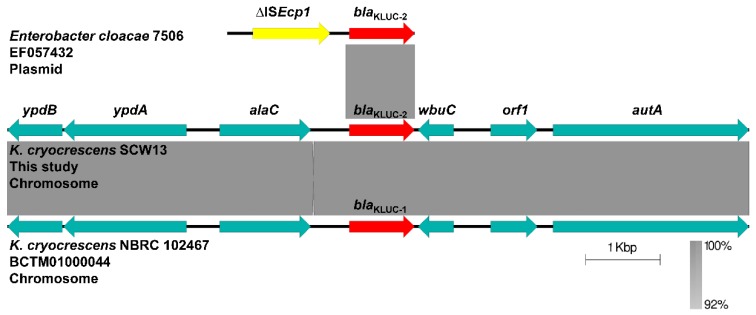
Comparative analysis of the genetic context of *bla*_KLUC-2_ and *bla*_KLUC-1_. Genes and insertion sequences are indicated by arrows. Light gray shades denote shared regions with a high degree of homology. The construction of sequence comparison was performed using BLAST [29] and Easyfig version 2.2.3.

In the process of genome sequence analysis, we found a *bla*_CTX-M-1_-like gene on the chromosome of SCW13, which was not identified by ResFinder. BLASTp analysis using the amino acid sequence of the *bla*_CTX-M-1_-like gene as a query matched (100% query coverage and 100% identity) KLUC-2 from *E. cloacae* 7506 in France in 2008. Analysis of the genetic environment of *bla*_KLUC-2_ showed that, in SCW13, *bla*_KLUC-2_ was sedentarily located on the chromosome, whereas *bla*_KLUC-2_ was located downstream of an IS*Ecp1* transposase on a plasmid in *E. cloacae* 7506 (Figure 2). It was proposed that the IS*Ecp1* element contributed to the mobilization of *bla*_KLUC-2_ from the *K. cryocrescens* chromosome to plasmids [19,26,27,28]. In this light, *bla*_KLUC-2_ in the *K. cryocrescens* strain SCW13 is most likely to be the chromosomal counterpart of plasmid-born *bla*_KLUC-2_. Besides, the genetic context of chromosomal *bla*_KLUC-2_ was identical to that of *bla*_KLUC-1_ on the chromosome of *K. cryocrescens* NBRC 102467 (Figure 2), suggesting that *bla*_KLUC-2_ may have evolved from *bla*_KLUC-1_ via a point mutation (G352→A)—or the other way around. The lack of more genetic information of *bla*_KLUC-2_ on plasmids was a limitation in this comparative analysis.

### 2.5. Phylogenetic Analysis of Different Kluyvera Species

**Figure 3:**
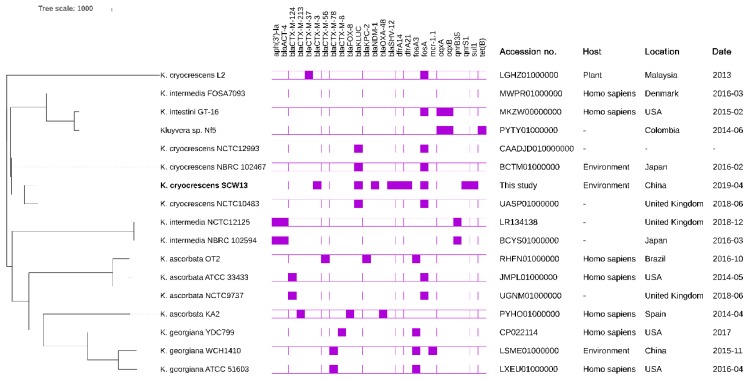
A phylogenetic analysis of the core genomes of *K. cryocrescens* strain SCW13 identified in this study (marked in bold) and 15 *Kluyvera* genomes deposited in the GenBank database (last accessed July 12, 2019). From left to right: (**1**) A maximum likelihood tree of *Kluyvera spp* strains. The phylogeny was inferred from the recombination-filtered single nucleotide polymorphism (SNP) alignment obtained by aligning a genome of *Kluyvera* isolate against the complete genome of *K. cryocrescens* NBRC 102467. (**2**) A heatmap of the antimicrobial resistance genes as determined by ABRicate. The presence or absence of antibiotic resistance genes is indicated by filled or empty squares, respectively. (**3**) The annotation of each *Kluyvera* isolate, including GenBank accession no., hosts of isolates, locations, and collection dates. -, not available.

A phylogenetic tree (Figure 3) was inferred based on 36,734 qualified SNPs. We found that SCW13 was closely related to *K. cryocrescens* NCTC10483, which was isolated in the United Kingdom in 2018. Five *K. cryocrescens* strains were included in the phylogenetic analysis, and they were tightly clustered with each other with the exception of a distinct member, *K. cryocrescens* L2. We suggested that *K. cryocrescens* L2, in fact, may not be a member of *K. cryocrescens*. Similarly, the *K. intermedia* strain FOSA7093 showed a greater evolutionary relatedness with *K. intestini* strain GT-16, but was less related to other *K. intermedia* members. We suggested that *K. intermedia* FOSA7093 should be recategorized as a *K. intestini* isolate. Thus, a taxonomic re-evaluation of the *Kluyvera* genus is required.

## 3. Materials and Methods

### 3.1. Strain Identification

*K. cryocrescens* strain SCW13 was recovered from the influx mainstream of hospital sewage at the affiliated hospital of Southwest Medical University, Luzhou in Western China, in April 2019. A volume of 10 mL of collected water was concentrated by centrifugation. The sediment was resuspended in 100 μL of Luria–Bertani broth culture and spread onto McConkey agar plates containing 1 μg/mL meropenem. Species identification was performed by sequencing of the 16S rRNA gene amplified with the universal primers 27F and 1492R [30].

### 3.2. Antimicrobial Susceptibility Testing

To examine the phenotypic resistance profile of SCW13, minimum inhibitory concentrations (MICs) of meropenem, imipenem, colistin, ceftriaxone, cefotaxime, cefoxitin, aztreonam, ciprofloxacin, gentamycin, amikacin, and tigecycline against the strain were determined using the microdilution broth method following the recommendations of the Clinical Laboratory Standards Institute (CLSI) [31]. The breakpoints of colistin and tigecycline were interpreted according to the European Committee on Antimicrobial Susceptibility Testing (EUCAST) (http://www.eucast.org/). Otherwise, we applied those defined by the CLSI.

### 3.3. Conjugation Assay

Conjugation experiments were carried out in broth using azide-resistant *E. coli* J53 as the recipient strain at 37 °C. Bacteria were spread on Luria–Bertani agar plates containing 150 μg/mL sodium azide plus 1 μg/mL meropenem, for selecting the transconjugants carrying *bla*_NDM-1_. The presence of *bla*_NDM-1_ in transconjugants was further confirmed by PCR and sequencing.

### 3.4. Genome Sequencing and Bioinformatic Analysis

Total genomic DNA of SCW13 was extracted using a Rapid Bacterial Genomic DNA Isolation Kit (Sangon Biotech, Shanghai, China). Purified DNA was subjected to whole genomic sequencing on the Illumina HiSeq 2000 system with the 150-bp paired-end approach and 100 × coverage. Reads were trimmed using Trimmomatic [32] and assembled using the Spades program [33]. Annotation was carried out using Prokka [34]. Genome-based species identification was performed by ANI analysis using the web program ANI Calculator [35].

Plasmids were completely circularized using PCR and Sanger sequencing to fill in the gaps between contigs. The plasmid replicon type was determined using the PlasmidFinder [36]. Annotations of the plasmid sequences were conducted using Prokka combined with BLASTp/BLASTn searches against the NCBI database. Antimicrobial resistance genes carried by plasmids were identified using the online database ResFinder [37]. Multiple and pairwise sequence comparisons were performed using BLAST and visualized with Easyfig v 2.2.3 [38].

Core genomes for *Kluyvera* strains (*n* = 16, accessed on 12 July, 2019) available in the GenBank database were aligned with that of the *K. cryocrescens* reference strain NBRC 102467 (Accession no. BCTM01000000) using CSI Phylogeny 1.4 [39]. Gubbins (version 2.3.4) was used to remove single nucleotide polymorphisms (SNPs) on recombination sites [40]. The filtered SNPs were used as input for inferring a phylogenetic tree using RAxML with the GTRGAMMA model and 1000 bootstraps [26]. Antimicrobial resistance genes in these genomes were identified using ABRicate (https://github.com/tseemann/abricate).

### 3.5. Nucleotide Sequence Accession Numbers

A draft genome sequence of SCW13 has been deposited into GenBank under the accession no. VKGH00000000. The complete sequences of pNDM1_SCW13, pSCW13-1 and pSCW13-2 have been deposited into GenBank under accession no. MN178638, MN178639 and MN178640, respectively.

## 4. Conclusions

In summary, we reported a *bla*_NDM−1_-carrying *K. cryocrescens* isolate for the first time. *bla*_NDM−1_ was carried by a pNDM-HN380-like IncX3 plasmid in this *K. cryocrescens* strain, which is a worrying development, as it highlights that *bla*_NDM−1_ has further spread to uncommon *Enterobacteriaceae* strains by epidemic IncX3 plasmids. Therefore, extensive surveillance and effective actions are urgently needed to control the spread of *bla*_NDM_-encoding IncX3 plasmids. In addition, our work also identified a *bla*_KLUC-2_ gene on the chromosome of *K. cryocrescens* SCW13, which represents the origin of the plasmid-mediated resistance gene *bla*_KLUC-2_.

## Figures and Tables

**Table 1:** Minimum inhibitory concentrations (MICs) for the *K. cryocrescens* strain SCW13, its transformant, and the recipient strain J53.

Strain	MIC (μg/mL) ^a^											
	AMK	FOS	GEN	CST	MEM	IMP	CEF	CFT	AZT	CIP	CTX	TGC
SCW13	32	256	≤4	≤4	128	256	>512	256	512	≤4	>512	≤4
SCW13 ^b^	16	64	≤4	≤4	128	256	>512	512	512	≤4	>512	≤4
*E. coli* J53	16	32	≤4	≤4	0.5	1	≤4	4	8	≤4	8	≤4

^a^ AMK, amikacin; FOS, fosfomycin; GEN, gentamicin; CST, colistin; MEM, meropenem; IMP, imipenem; CEF, ceftriaxone; CFT, cefoxitin; AZT, aztreonam; CIP, ciprofloxacin; CTX, cefotaxime; TGC, tigecycline. Resistance is highlighted in bold. ^b^ *E. coli* J53 transformant.
